# Maternal periodontitis may cause lower birth weight in children: genetic evidence from a comprehensive Mendelian randomization study on periodontitis and pregnancy

**DOI:** 10.1007/s00784-024-05591-9

**Published:** 2024-03-05

**Authors:** Xixiong Chen, Xiao Li, Kun Yang, Jinlin Fang

**Affiliations:** 1 Department of Stomatology, First people’s Hospital of Linping District, No. 365 Yingbing Road, Hangzhou, 310000 China; 2 Department of Stomatology, First people’s Hospital of Yuhang District, Hangzhou, China; 3 Department of Nursing, Shaoxing Seventh people’s Hospital, Shaoxing, China

**Keywords:** Periodontitis, Spontaneous miscarriage, Birth weight, Mendelian randomization

## Abstract

**Objectives:**

This study aims to comprehensively investigate the potential genetic link between periodontitis and adverse pregnancy outcomes using a two-sample Mendelian Randomization approach.

**Materials and methods:**

We employed robust genetic instruments for chronic periodontitis as exposure data from the FinnGen database. Data encompassing various pregnancy stage outcomes, including pre-pregnancy conditions (irregular menstruation, endometriosis, abnormal reproductive bleeding, and female infertility), pregnancy complications (hemorrhage, spontaneous miscarriage, and abnormalities in products), and post-pregnancy factors (single spontaneous delivery, labor duration, and birth weight of the child), were obtained from the UK Biobank. The random-effects inverse-variance weighted (IVW) method was utilized to compute primary estimates while diligently assessing potential directional pleiotropy and heterogeneity.

**Results:**

Our findings indicate a negative association between periodontitis and labor duration (odds ratio [OR] = 0.999; 95% confidence interval [CI]: 0.999 to 1.000; *P* = 0.017). Individuals with periodontitis are more likely to deliver lower-weight infants (OR = 0.983; 95% CI: 0.972 to 0.995; *P* = 0.005). We found no evidence of pleiotropy or heterogeneity in aforementioned two associations. We did not observe casual links with pre-pregnancy conditions and pregnancy complications.

**Conclusions:**

This Mendelian Randomization study underscores the genetic influence of periodontitis on specific adverse pregnancy outcomes, particularly concerning labor duration and lower birth weight deliveries.

**Clinical relevance:**

Our study emphasizes the critical importance of maintaining periodontal health during pregnancy and offers genetic evidence supporting these associations. Further investigation is required to delve deeper into the specific underlying mechanisms.

**Supplementary Information:**

The online version contains supplementary material available at 10.1007/s00784-024-05591-9.

## Introduction

Periodontitis is a chronic inflammatory condition that targets the tissues surrounding the teeth, resulting in gum recession, tooth mobility, and potential tooth loss [1]. Epidemiological surveys by reveal that in China, 63.3% of individuals suffer from periodontal disease, with 30.6% experiencing severe periodontitis (stage III or IV) [2]. Globally, the prevalence of periodontitis is estimated at around 62%, with severe periodontitis affecting 23.6% [3].

Furthermore, beyond its impact on periodontal tissues, periodontitis also has implications for other bodily systems. Some observational studies have shown that periodontitis may pose potential risks for female reproductive difficulties and adverse pregnancy outcomes, such as female infertility [4], endometriosis [5], spontaneous miscarriages occurring before 20 weeks of gestation [6], and the delivery of low-weight babies, defined as those weighing less than 2.5 kg at birth [7, 8].

Given the relatively high incidence of periodontitis, global concern of female reproductive difficulties [9] and adverse pregnancy outcomes [10], combined with the reality that that periodontitis can be significantly improved and controlled, so it is crucial to thoroughly investigate the relationship between periodontitis and pregnancy.

However, establishing a definitive causal relationship between periodontitis and some pregnancy difficulties and outcomes remains challenging due to confounding risks and measurement errors. Ethical concerns and the extended duration required for high-quality randomized clinical trials further complicated matters. To shed light on the causal nature of the effect of periodontitis on pregnancy, Mendelian Randomization (MR) has emerged as a promising strategy. This approach utilizes summary statistics from large-scale genome-wide association studies (GWAS).

In our research, we aim to comprehensively explore the impact of periodontitis throughout pregnancy, considering various outcomes at different stages. We have selected irregular menstruation, endometriosis, abnormal reproductive bleeding, and female infertility as pre-pregnancy outcomes, as these factors can significantly influence the success rate of pregnancy [11]. During pregnancy, we will examine outcomes such as hemorrhage, spontaneous miscarriage, and abnormalities in products of conception. After pregnancy, we will assess single spontaneous delivery, labor duration, and the birth weight of the child as outcomes during this stage.

## Materials and methods

### Assumptions

**Fig. 1:**
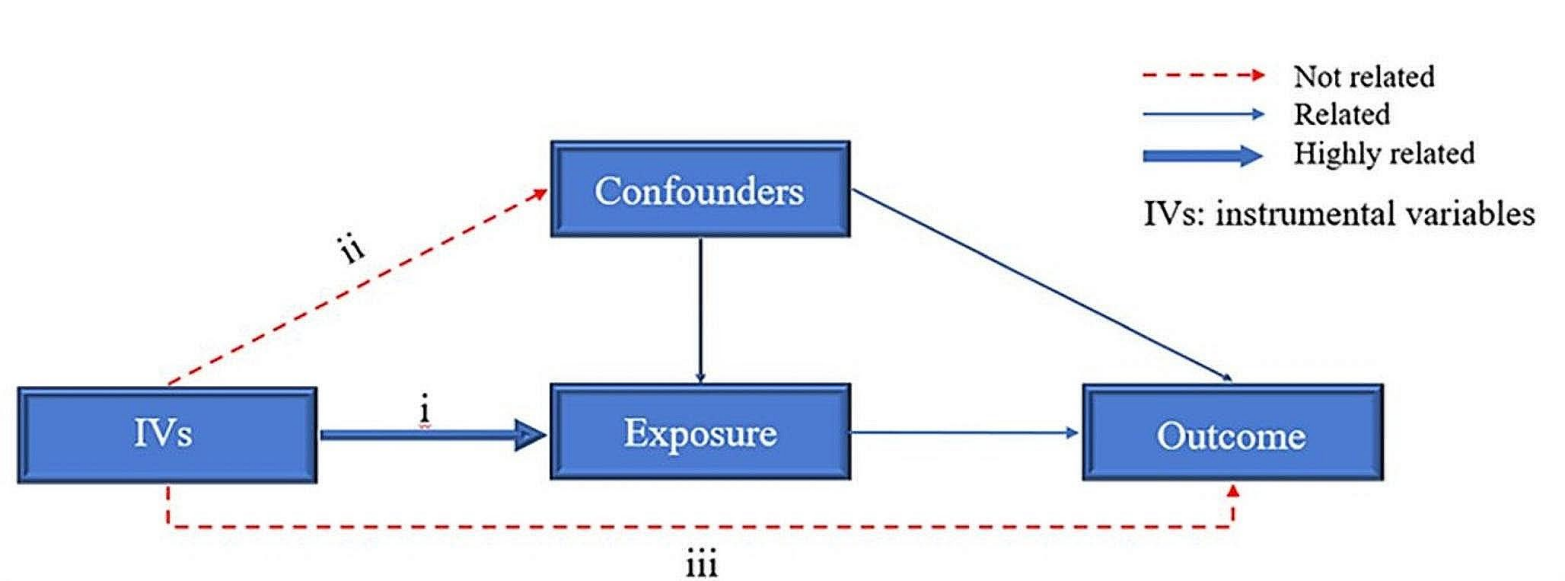
The basic assumptions of Mendelian randomization

A robust MR estimate is based on three fundamental assumptions [12]: (i) the instrumental variables (IVs) have a strong association with the exposure; (ii) the IVs are free from confounding factors; (iii) the IVs influence the outcomes solely through their impact on the exposure and not through an alternative causal pathway(Fig. 1).

### Data sources

To avoid sample overlapping [13], the data for exposure and outcomes were sourced from two completely separate consortia. The genome-wide association summary statistics (GWAS) related to chronic periodontitis were obtained from the R9 version of the FinnGen website (https://r9.finngen.fi/), encompassing 263,668 samples (4,434 cases and 259,234 controls). Chronic periodontitis, as defined by this database (https://risteys.finregistry.fi/endpoints/K11_PERIODON_CHRON), encompasses patients diagnosed with the condition from hospitals, exhibiting both pronounced and complex forms. Clinical manifestations include attachment loss, deep periodontal pockets, alveolar bone loss, and other relevant features. All data for various outcomes are from the UK Biobank (UKBB)(http://www.nealelab.is/uk-biobank). It’s worth noting that a portion of the data in the UKBB database originates from finngen sources, so we have ensured not to utilize it as outcome data. All samples are from European populations. Detailed sample information is provided in Table 1.

**Table 1:** The detail of data information

Phenotype	Consortium	Source	Variable type	Sample size	ncase	ncontrol
Chronic periodontitis	FinnGen	finngen	categorical	263,668	4434	259,234
Excessive, frequent and irregular menstruation	UKBB	ICD-10	categorical	361,194	8475	352,719
Abnormal uterine and vaginal bleeding	UKBB	ICD-10	categorical	361,194	2455	358,739
Endometriosis	UKBB	ICD-10	categorical	361,194	1496	359,698
Female infertility	UKBB	ICD-10	categorical	361,194	696	360,498
Hemorrhage in early pregnancy	UKBB	ICD-10	categorical	361,194	738	360,456
Other abnormal products of conception	UKBB	ICD-10	categorical	361,194	1106	360,088
Number of spontaneous miscarriages	UKBB	phesant	ordinal	60,300	/	/
Single spontaneous delivery	UKBB	ICD-10	categorical	361,194	1672	359,522
Long labour	UKBB	ICD-10	categorical	361,194	1060	360,134
Birth weight of the first child	UKBB	phesant	ordinal	155,202	/	/

Ordinal data statement: Number of spontaneous miscarriages [score1: 0; score 2: 1; score 3:>1]; Birth weight of the first child [score1:<7 pounds; score 2: =7 pounds; score 3:>7 pounds]

### Selection of genetic instrumental variables

A qualified genetic instrument [14] should demonstrate a robust association with the exposure (*p*<5 × 10^− 6^). Additionally, it should exhibit no significant linkage disequilibrium [LD] (r2 < 0.001) even when considering a window size of 10 MB. Single Nucleotide Polymorphisms (SNPs) that exhibited significant associations with the outcome (*p* < 5 × 10^− 8^) were excluded. The strength of the selected SNPs should be evaluated by calculating each SNP’s F-statistic. Only those SNPs with F> 10 are eligible for subsequent analysis using the following formulas [15, 16]:

$$F=\frac{{R}^{2}}{1-{R}^{2}}\times \frac{N-K-1}{K}$$

$${R}^{2}=2\times MAF\times \left(1-MAF\right)\times {beta}^{2}$$

To explore the presence of horizontal pleiotropy, we conducted MR Pleiotropy RESidual Sum and Outlier (MR PRESSO) analysis, with particular attention to any outliers in cases where horizontal pleiotropy was identified in less than 50% of the instruments [17]. To further validate our selected SNPs and ensure their independence from the outcome, we employed PhenoScanner [18] to find any SNPs to be associated with the outcome and then were systematically removed from our analysis.

### Mendelian randomization analyses

**Fig. 2:**
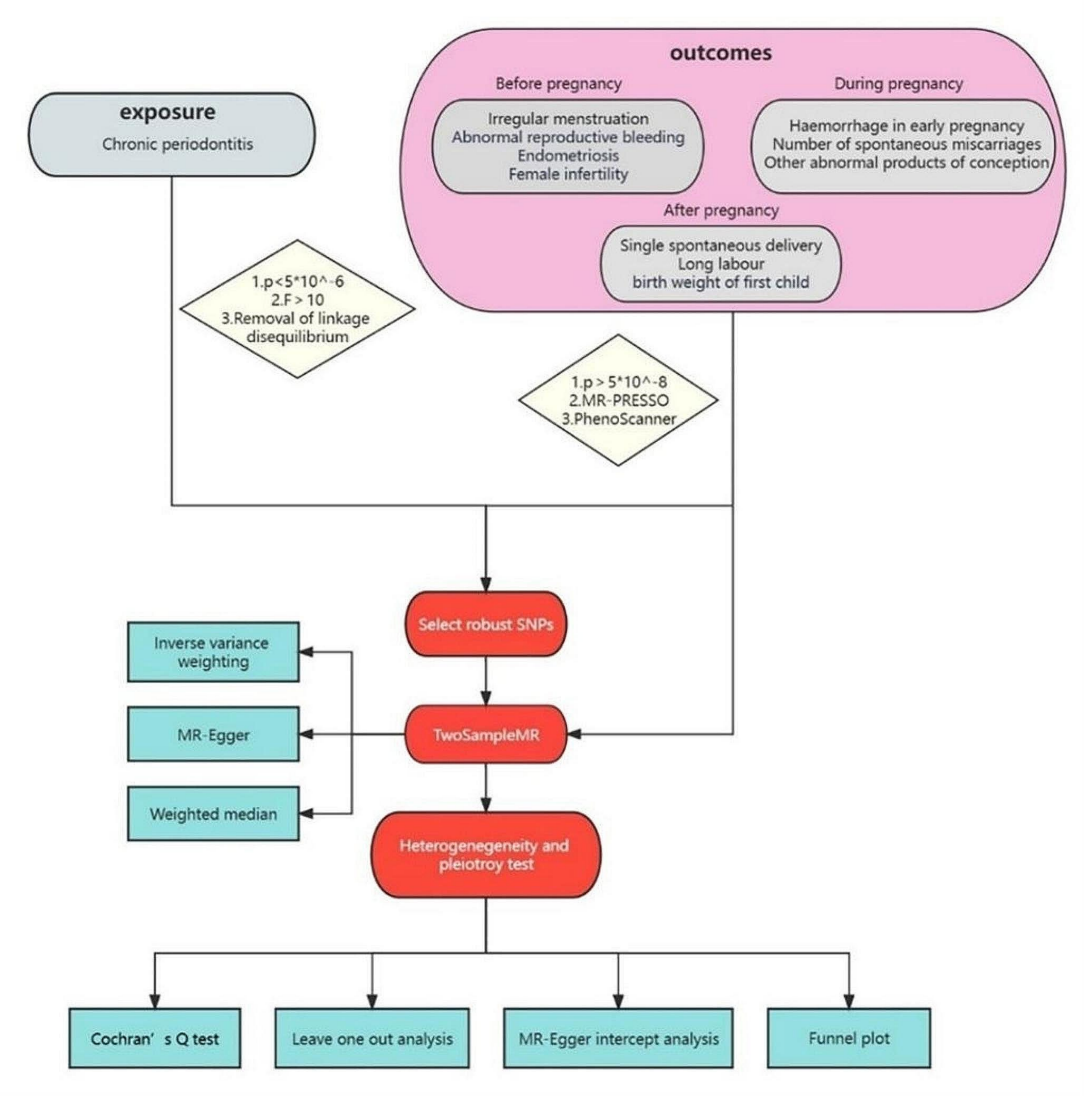
The study frame chart of Mendelian randomization study

Three distinct MR methods, including random-effects inverse-variance weighted (IVW), MR Egger, and weighted median, were employed to assess the causal relationship between exposure and outcomes. In general, the primary results were derived from the IVW method, which combined the Wald ratio of each SNP on the outcome to obtain a pooled causal estimate. The results obtained through the other two methods can be considered supplementary to IVW. These complementary methods enhance the robustness of MR results across a broader spectrum of scenarios. The MR-Egger method is assumed that the instrument’s strength is independent of the direct effect of the instrument on the outcome, even in the presence of pleiotropy [19]. Conversely, the weighted median method can be reliably estimated when at least half of the weighted variance introduced by horizontal pleiotropy is valid [20]. For significant estimates, we conducted MR-Egger intercept test to examine horizontal pleiotropy. We also implemented leave-one-out analyses to scrutinize potential outliers. Additionally, the Cochran’s Q test was utilized to detect any significant heterogeneity in the results. Furthermore, we employed a funnel plot as a visual tool to assess the possibility of directional pleiotropy. This comprehensive approach allowed us to delve deeper into the potential sources of bias and provide a more robust evaluation of our findings. R software (version 4.3.1) and R Package “TwoSampleMR” and “MRPRESSO” were used to conduct all statistical analyses in the MR analysis. The study frame chart is presented in Fig. 2.

## Results

**Fig. 3:**
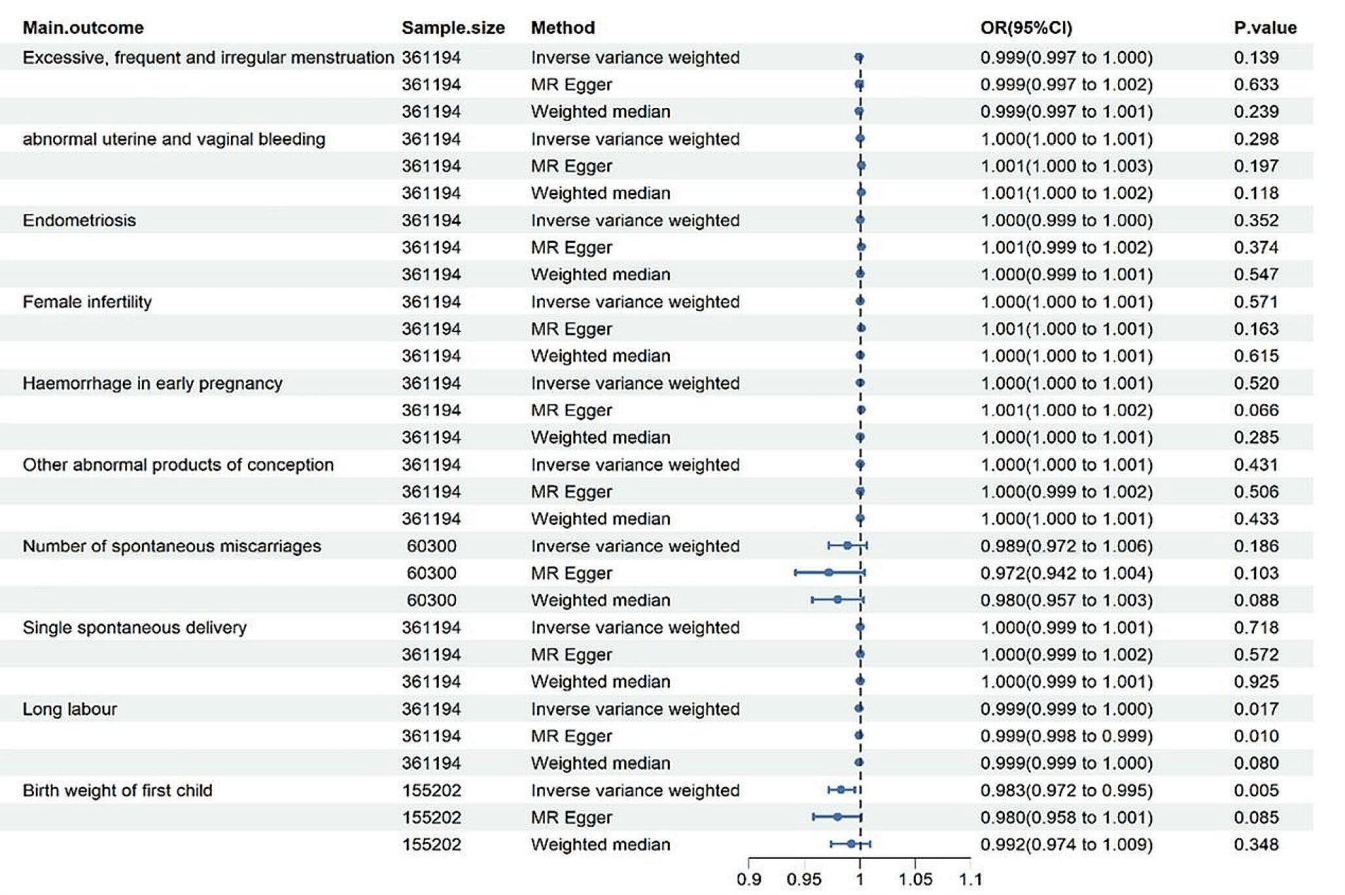
Forest plot summarizing the MR results

In total, 17 index SNPs were selected for the genetic prediction of chronic periodontitis. All of their F-statistics ranged from 43 to 124 to ensure the robustness of the instrumental variables (IVs) (Supplementary Material 1). We conducted a comprehensive Mendelian Randomization (MR) study on periodontitis concerning pregnancy. The causal effect of IVW was used as the main result. The MR results were presented in Fig. 3.

In our research, we found no genetic association between periodontitis and certain pregnancy difficulties, including excessive, frequent, and irregular menstruation (OR = 0.999, 95% CI: 0.997 to 1.001, *P* = 0.139), abnormal uterine and vaginal bleeding (OR = 1.000, 95% CI: 1.000 to 1.001, *P* = 0.298), Endometriosis (OR = 0.999, 95% CI: 0.999 to 1.000, *P* = 0.352), and female infertility (OR = 1.000, 95% CI: 1.000 to 1.001, *P* = 0.571) (Fig. 3). The results of the Cochran‘s Q test showed no heterogeneity(Table 2). No outliers were identified by MR PRESSO (Table 2), funnel plots (Supplementary Material 2), and leave-one-out plots (Supplementary Material 3) concerning the relationship between periodontitis and the aforementioned outcomes. MR Egger intercept tests also rejected the presence of horizontal pleiotropy (Table 2).

During pregnancy, we evaluated the effects of periodontitis on outcomes such as hemorrhage in early pregnancy, other abnormal products of conception, and the number of spontaneous miscarriages. Heterogeneity was observed when we selected other abnormal products of conception as the outcome (Cochran’s Q = 26.79, P_Cochran’s Q_=0.044). However, the results were acceptable because we employed the random-effects IVW method [21]. There was no genetic association found between periodontitis and hemorrhage in early pregnancy (OR = 1.000, 95% CI: 1.000 to 1.001, *P* = 0.520), other abnormal products of conception (OR = 1.000, 95% CI: 1.000 to 1.001, *P* = 0.431) and occurrence of spontaneous miscarriages (OR = 0.989, 95% CI: 0.972 to 1.006, *P* = 0.186). Furthermore, there was no evidence of pleiotropy between periodontitis and outcomes during pregnancy, as indicated by MR PRESSO tests, MR Egger intercept tests (Table 2), funnel plots (Supplementary Material 2),and leave-one-out plots (Supplementary Material 3).

After pregnancy, we assessed the effects of periodontitis on delivery and the weight of newborns. There was no genetic correlation observed between periodontitis and single spontaneous delivery (OR = 1.000, 95% CI: 0.999 to 1.001, *P* = 0.718). Surprisingly, periodontitis was associated with a reduction in labor duration (OR = 0.999, 95% CI: 0.999 to 1.000, *P* = 0.017). The weak association observed in our study is consistent with the results obtained through MR Egger (OR = 0.999, 95% CI: 0.998 to 0.999, *P* = 0.010). For the birth weight of the first child, women with periodontitis appeared to deliver babies with lower birth weight (OR = 0.983, 95% CI: 0.972 to 0.995, *P* = 0.005). There was no significant evidence of pleiotropy and heterogeneity between periodontitis and single spontaneous delivery, labor duration, and birth weight of the first child (Table 2) , (Supplementary Material 2, 3).

**Table 2:** Summary of heterogeneity and multiplicity results

Outcomes	Cochran’s Q	P_Cochran’s Q_	MR Egger intercept	P_MR Egger intercept_	Global test RSSobs	P_Global_
Excessive, frequent and irregular menstruation	8.552	0.931	-9.90 × 10^− 05^	0.728	10.748	0.929
abnormal uterine and vaginal bleeding	12.35	0.720	-1.45 × 10^− 04^	0.363	13.946	0.817
Endometriosis	21.905	0.146	-2.26 × 10^− 04^	0.110	26.495	0.135
Female infertility	9.479	0.892	-1.12 × 10^− 04^	0.189	11.418	0.917
Hemorrhage in early pregnancy	10.44	0.843	-1.63 × 10^− 04^	0.072	11.935	0.872
Other abnormal products of conception	26.79	**0.044**	-4.40 × 10^− 05^	0.751	30.194	0.071
Number of spontaneous miscarriages	14.601	0.554	3.94 × 10^− 03^	0.241	17.801	0.524
Single spontaneous delivery	19.205	0.258	-1.25 × 10^− 04^	0.378	26.243	0.119
Long labour	8.981	0.914	1.99 × 10^− 04^	0.065	11.093	0.911
Birth weight of first child	10.066	0.863	8.78 × 10^− 04^	0.698	23.116	0.265

Abbreviations: RSSobs [observed residual sum of squares]

## Discussion

To the best of our knowledge, this is the first comprehensive MR study aimed at evaluating the causal relationship between periodontitis and pregnancy, as well as delivery outcomes. Our primary objective was to assess whether periodontitis, from a genetic perspective, increases the risk of pregnancy difficulties (including abnormal menstruation, abnormal reproductive bleeding, endometriosis, and female infertility), leads to adverse pregnancies (involving hemorrhage in early pregnancy, abnormalities in products of conception, and spontaneous miscarriages), and affects delivery outcomes (such as single spontaneous delivery, labor duration, and the birth weight of the first child).

To mitigate potential bias, we took several relevant actions. First, we ensured that the samples for exposure and outcomes were deprived from completely separate European populations, thus effectively eliminating issues related to sample overlapping, which can lead to Type 1 errors and spurious trait associations [22]. Second, we rigorously selected robust genetic instruments with F-statistics exceeding 40, signifying their high relevance to chronic periodontitis. Third, we utilized a variety of methods, including the leave-one-out test, MR-PRESSO analysis, MR Egger intercept analysis, and funnel plots, to address directional pleiotropy and exclude any abnormal outliers.

Only by following these rigorous steps can we accurately interpret the results. As for the outcomes, we identified causal relationships between periodontitis and labor duration, as well as birth weight.

Previous studies have presented controversial evidence from observational studies [23, 24] and/or potential underlying mechanisms from some laboratory studies [10, 25] regarding the link between periodontitis and birth weight in newborns as well as labor time. However, both observational and laboratory studies have limitations, including confounding biases, small sample sizes, contingencies, and species differences. MR can leverage human genetic data to investigate these effects, effectively overcoming limitations by utilizing robust SNPs from large databases. Our genetic analysis revealed a negative correlation between maternal periodontitis and both labor duration and birth weight in newborns, providing genetic evidence supporting the notion that maternal periodontitis may increase the risk of low-weight infants. This also underscores the need for future research exploring the association between periodontitis and labor duration.

The natural labor process initiates with regular uterine contractions and concludes when the baby, placenta, and membranes are delivered [26]. Our research shows a subtle negative genetic association [27] between periodontitis and labor duration, determined by the random-effect IVW method (OR = 0.999, 95% CI: 0.999 to 1.000, *P* = 0.017) and the MR Egger method (OR = 0.999, 95% CI: 0.998 to 0.999, *P* = 0.010)(Fig. 3). These subtle effects warrant further validation with additional positive cases.

Regrettably, there is a dearth of observational studies investigating the relationship between periodontitis and labor duration. This discrepancy may be attributed to the lack of universal or standardized definitions of normal labor duration [28]. Nevertheless, there are plausible explanations for this connection. Some studies [29, 30] have confirmed intrauterine colonization with oral microbes, even in clinically healthy pregnancies, which means that pathogenic bacteria from periodontal sources can colonize the uterus and have the potential to affect the fetus. Additionally, certain periodontal-origin inflammatory mediators, such as IL-2, IL-6, IL-10, TNF-α, and PGE-2, can initiate metabolic processes via the bloodstream [31]. IL-1, IL-6, and TNF‐α may stimulate the production of prostaglandins in the chorion, which can stimulate uterine contractions, cervical ripening, and accelerate the labor process [10].

Regarding low birth weight of newborns, defined as a weight less than 2.5 kg at birth, numerous observational studies [23, 32] have highlighted the risk of periodontitis for pregnant women delivering low-weight children. In our research, we used ordinal data for the weight of the first child as the outcome. One disadvantage of this type of data is that, unlike continuous variables that enable precise analysis, the database categorizes weight into three levels (< 7 pounds, = 7 pounds, > 7 pounds), which lacks granularity and impedes the ability to analyze the extent to which maternal periodontitis reduces children’s weight. The correct interpretation of ordinal results [33] suggests that pregnant women with periodontitis may deliver children with lower-weight level *(OR = 0.983, 95% CI: 0.972 to 0.995, P = 0.005)*. Although our weight classification (7 pounds ≈ 3.175 kg) does not precisely align with the standard for low birth weight (2.5 kg), our research sheds light on the causal effect of periodontitis on birth weight, emphasizing the importance of maintaining maternal periodontal health for infant well-being.

This finding is further supported by numerous laboratory studies. These studies [34–36] have detected various periodontal pathogens, such as *Fusobacterium nucleatum*, *Porphyromonas gingivalis*, *Campylobacter rectus*, *Tannerella forsythia*, *Prevotella nigrescens*, and *Streptococcus mitis* in the amniotic fluid from mothers. Additionally, focal injury induced by *Campylobacter rectus* has been associated with a significant decrease in the size of the labyrinth layer, responsible for nutrient exchange between the mother and fetus. This suggests insufficient fetal nutrition, potentially justifying impaired growth [25]. Moreover, these placentas were linked to reduced expression of genes related to placental and fetal growth [37].

**Fig. 4:**
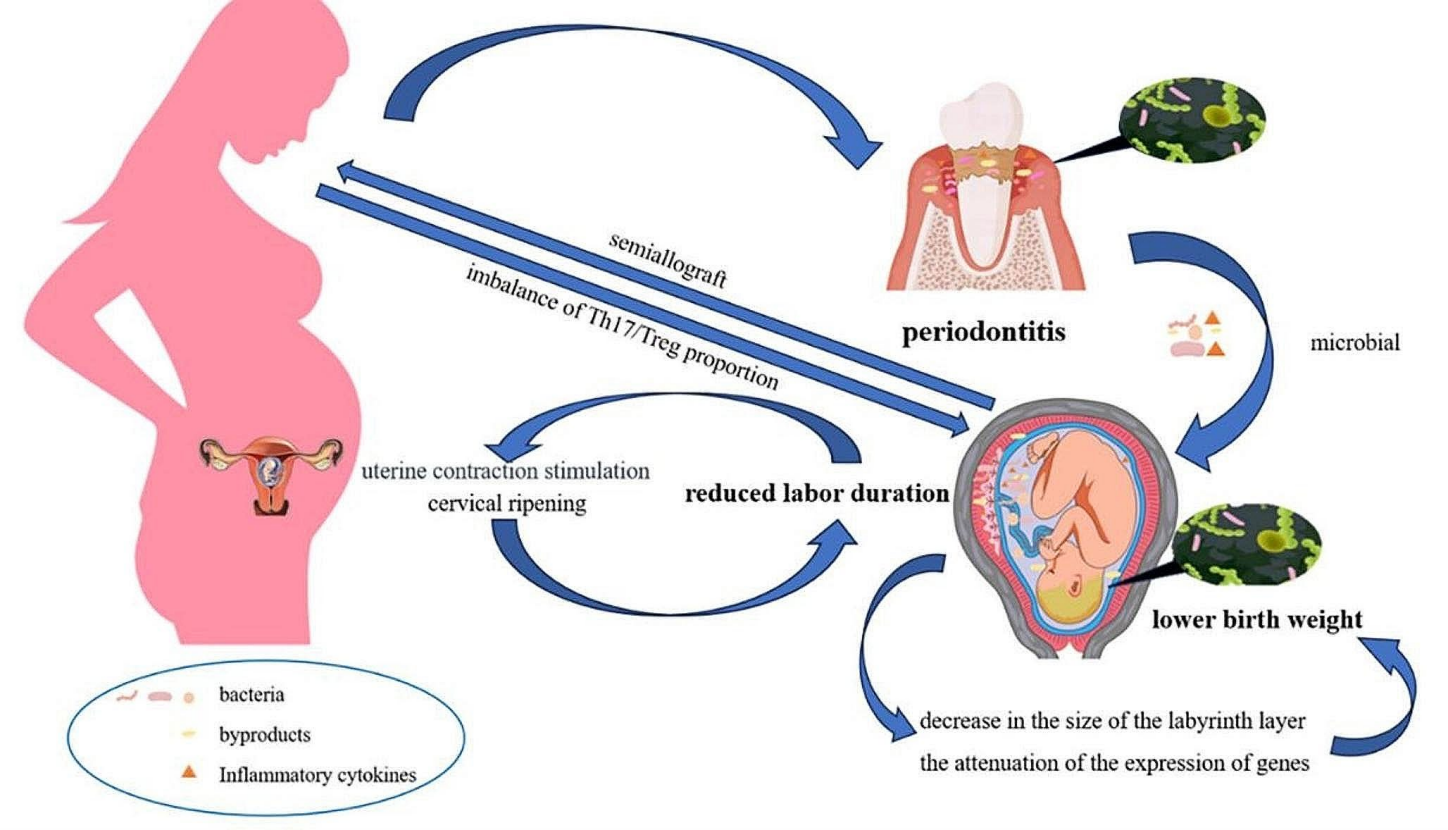
Biological mechanism of maternal periodontitis resulting in reduced labour duration and lower birth weight child

Beyond focal infection, the maternal immune system also plays a crucial role. As a semi-allograft, the fetus carries external DNA from the father, which must be tolerated by the mother throughout pregnancy [38]. Unfortunately, periodontal microbe infections trigger a shift in the maternal immune response toward a pathogenic inflammatory response, disrupting the maternal-fetal interface’s homeostasis [39]. Some infectious diseases may disturb the Th17/Treg proportion, leading to increased Th1/Th17 cell numbers and activity. The Th1 response activates decidual macrophages, which release excessive TNF-α and nitric oxide, detrimental to the fetus [39, 40]. The potential biological mechanism is depicted in Fig. 4.

However, several aspects remain unexplained within this theory. Firstly, there is insufficient evidence to determine which periodontal treatment is superior in preventing adverse obstetric outcomes in some studies [41, 42]. Secondly, the current evidence does not provide answers as to why some women develop adverse pregnancy outcomes while others do not, despite concurrent bacterial colonization [43]. Thirdly, our study does not currently support a link between periodontitis and an increased rate of spontaneous abortion (Fig. 3).

There are also several limitations associated with the MR method. Primarily, it necessitates a large sample size, particularly for exposures, as this determines the number and quality of IVs [44]. Therefore, we refrain from conducting reverse MR to avoid issues related to the directionality of these associations (ncase_periodontitis_=458, UK Biobank). While funnel plots are used for scrutinizing the exclusion of inverse associations, they may involve some subjectivity. Additionally, unknown confounders that have not been excluded could also impact the stability of this association due to fundamental assumptions (Fig. 1). Finally, our study has exclusively delved into this association within the confines of European populations, with no certainty regarding its prevalence in other geographical regions.

Given the constraints imposed by our available data, our findings furnish genetic evidence, opening doors for researchers to investigate the ramifications of periodontitis on adverse pregnancy outcomes, specifically focusing on labor duration and birth weight. Subsequent research endeavors should strive to unravel the precise mechanisms underpinning these effects and seek effective therapeutic strategies to avert adverse outcomes in individuals afflicted by periodontitis.

### Electronic supplementary material

Below is the link to the electronic supplementary material.

Supplementary Material 1

Supplementary Material 2

Supplementary Material 3

## References

[1] Slots J (2017) Periodontitis: facts, fallacies and the future. Periodontol 2000 75(1):7–23. 10.1111/prd.12221. PubMed PMID: 28758294 10.1111/prd.12221 28758294

[2] Jiao J, Jing W, Si Y, Feng X, Tai B, Hu D (2021). The prevalence and severity of periodontal disease in Mainland China: data from the Fourth National oral health survey (2015–2016). J Clin Periodontol.

[3] Trindade D, Carvalho R, Machado V, Chambrone L, Mendes JJ, Botelho J (2023). Prevalence of periodontitis in dentate people between 2011 and 2020: a systematic review and meta-analysis of epidemiological studies. J Clin Periodontol.

[4] Yildiz Telatar G, Gurlek B, Telatar BC (2021). Periodontal and caries status in unexplained female infertility: a case-control study. J Periodontol.

[5] Kavoussi SK, West BT, Taylor GW, Lebovic DI (2009). Periodontal disease and endometriosis: analysis of the National Health and Nutrition Examination Survey. Fertil Steril.

[6] Bond JC, Wise LA, Fox MP, Garcia RI, Murray EJ, White KO (2023). Preconception Periodontitis and risk of spontaneous abortion in a prospective cohort study. Am J Epidemiol.

[7] Bhavsar NV, Trivedi S, Vachhani KS, Brahmbhatt N, Shah S, Patel N (2023). Association between preterm birth and low birth weight and maternal chronic periodontitis: a hospital-based case-control study. Dent Med Probl.

[8] Hussain V, Waseem A, Husain I, Waseem U, Shahbaz M, Qureshi F (2023). The Association of Periodontal Disease with Low Birth Weight infants: a Case Control Study. Matern Child Health J.

[9] Wang Y, Fu Y, Ghazi P, Gao Q, Tian T, Kong F (2022). Prevalence of intimate partner violence against infertile women in low-income and middle-income countries: a systematic review and meta-analysis. Lancet Glob Health.

[10] Bobetsis YA, Graziani F, Gursoy M, Madianos PN (2020). Periodontal disease and adverse pregnancy outcomes. Periodontol 2000.

[11] Venkatesh SS, Ferreira T, Benonisdottir S, Rahmioglu N, Becker CM, Granne I (2022). Obesity and risk of female reproductive conditions: a Mendelian randomisation study. PLoS Med.

[12] Skrivankova VW, Richmond RC, Woolf BAR, Davies NM, Swanson SA, VanderWeele TJ (2021).

[13] Burgess S, Davies NM, Thompson SG (2016). Bias due to participant overlap in two-sample Mendelian randomization. Genet Epidemiol.

[14] Kwok MK, Kawachi I, Rehkopf D, Schooling CM (2020). The role of cortisol in ischemic heart disease, ischemic stroke, type 2 diabetes, and cardiovascular disease risk factors: a bi-directional Mendelian randomization study. BMC Med.

[15] Palmer TM, Lawlor DA, Harbord RM, Sheehan NA, Tobias JH, Timpson NJ (2012). Using multiple genetic variants as instrumental variables for modifiable risk factors. Stat Methods Med Res.

[16] Pierce BL, Burgess S (2013). Efficient design for Mendelian randomization studies: subsample and 2-sample instrumental variable estimators. Am J Epidemiol.

[17] Verbanck M, Chen CY, Neale B, Do R (2018). Detection of widespread horizontal pleiotropy in causal relationships inferred from Mendelian randomization between complex traits and diseases. Nat Genet.

[18] Chen X, Kong J, Pan J, Huang K, Zhou W, Diao X (2021). Kidney damage causally affects the brain cortical structure: a Mendelian randomization study. EBioMedicine.

[19] Bowden J, Davey Smith G, Burgess S (2015). Mendelian randomization with invalid instruments: effect estimation and bias detection through Egger regression. Int J Epidemiol.

[20] Bowden J, Davey Smith G, Haycock PC, Burgess S (2016). Consistent estimation in Mendelian randomization with some Invalid instruments using a weighted median estimator. Genet Epidemiol.

[21] Bowden J, Del Greco MF, Minelli C, Davey Smith G, Sheehan N, Thompson J (2017). A framework for the investigation of pleiotropy in two-sample summary data Mendelian randomization. Stat Med.

[22] Hu X, Zhao J, Lin Z, Wang Y, Peng H, Zhao H (2022). Mendelian randomization for causal inference accounting for pleiotropy and sample structure using genome-wide summary statistics. Proc Natl Acad Sci U S A.

[23] Sinha A, Singh N, Gupta A, Bhargava T, Kumar PCM (2022). Relationship between the Periodontal status of pregnant women and the incidence and severity of pre-term and/or low Birth Weight deliveries: a retrospective observational case-control study. Cureus.

[24] Fogacci MF, Cardoso EOC, Barbirato DDS, de Carvalho DP, Sansone C (2018). No association between periodontitis and preterm low birth weight: a case-control study. Arch Gynecol Obstet.

[25] Offenbacher S, Riche EL, Barros SP, Bobetsis YA, Lin D, Beck JD (2005). Effects of maternal Campylobacter rectus infection on murine placenta, fetal and neonatal survival, and brain development. J Periodontol.

[26] Wei H, Guan Q, Yu Q, Chen T, Wang X, Xia Y (2022) Assessing maternal thyroid function and its relationship to duration of the first stage of labor. Eur Thyroid J 11(2). Epub 20220317. 10.1530/ETJ-21-0071. PubMed PMID: 35166213; PubMed Central PMCID: PMCPMC8963166 10.1530/ETJ-21-0071 PMC8963166 35166213

[27] Ma XS, Sun J, Geng R, Zhao Y, Xu WZ, Liu YH (2023). Statins and risk of venous thromboembolic diseases: a two-sample Mendelian randomization study. Nutr Metab Cardiovasc Dis.

[28] Abalos E, Oladapo OT, Chamillard M, Diaz V, Pasquale J, Bonet M (2018). Duration of spontaneous labour in ‘low-risk’ women with ‘normal’ perinatal outcomes: a systematic review. Eur J Obstet Gynecol Reprod Biol.

[29] Aagaard K, Ma J, Antony KM, Ganu R, Petrosino J, Versalovic J (2014). The placenta harbors a unique microbiome. Sci Transl Med.

[30] Stout MJ, Conlon B, Landeau M, Lee I, Bower C, Zhao Q et al (2013) Identification of intracellular bacteria in the basal plate of the human placenta in term and preterm gestations. Am J Obstet Gynecol 208(3):226 e1–7. Epub 20130117. 10.1016/j.ajog.2013.01.018. PubMed PMID: 23333552; PubMed Central PMCID: PMCPMC3740162 10.1016/j.ajog.2013.01.018 PMC3740162 23333552

[31] Latorre Uriza C, Velosa-Porras J, Roa NS, Quinones Lara SM, Silva J, Ruiz AJ, Escobar Arregoces FM. (2018) Periodontal, disease inflammatory cytokines, and PGE(2) in pregnant patients at risk of preterm delivery: a pilot study. Infect Dis Obstet Gynecol 2018:7027683. Epub 20180801. 10.1155/2018/7027683. PubMed PMID: 30154640; PubMed Central PMCID: PMCPMC6093048 10.1155/2018/7027683 PMC6093048 30154640

[32] Arima H, Calliope AS, Fukuda H, Nzaramba T, Mukakarake MG, Wada T (2022). Oral cleaning habits and the copy number of periodontal bacteria in pregnant women and its correlation with birth outcomes: an epidemiological study in Mibilizi, Rwanda. BMC Oral Health.

[33] Catalan A, Tognin S, Hammoud R, Aymerich C, Pedruzo B, Bilbao-Gonzalez A (2023). Understanding the relationship between time spent outdoors, mental well-being and health-related behaviours in a Spanish sample: a real time smartphone-based study. Psychiatry Res.

[34] Narita Y, Kodama H (2022) Identification of the specific microbial community compositions in saliva associated with periodontitis during pregnancy. Clin Oral Investig 26(7):4995–5005. Epub 20220329. 10.1007/s00784-022-04468-z. PubMed PMID: 35352183 10.1007/s00784-022-04468-z 35352183

[35] Chopra A, Radhakrishnan R, Sharma M (2020). Porphyromonas gingivalis and adverse pregnancy outcomes: a review on its intricate pathogenic mechanisms. Crit Rev Microbiol.

[36] Chaemsaithong P, Lertrut W, Kamlungkuea T, Santanirand P, Singsaneh A, Jaovisidha A (2022). Maternal septicemia caused by Streptococcus mitis: a possible link between intra-amniotic infection and periodontitis. Case report and literature review. BMC Infect Dis.

[37] Bobetsis YA, Barros SP, Lin DM, Arce RM, Offenbacher S (2010). Altered gene expression in murine placentas in an infection-induced intrauterine growth restriction model: a microarray analysis. J Reprod Immunol.

[38] La Rocca C, Carbone F, Longobardi S, Matarese G (2014) The immunology of pregnancy: regulatory T cells control maternal immune tolerance toward the fetus. Immunol Lett 162(1 Pt A):41–8. Epub 20140701. 10.1016/j.imlet.2014.06.013. PubMed PMID: 24996040 10.1016/j.imlet.2014.06.013 24996040

[39] Wen X, Fu X, Zhao C, Yang L, Huang R (2023). The bidirectional relationship between periodontal disease and pregnancy via the interaction of oral microorganisms, hormone and immune response. Front Microbiol.

[40] Zenclussen AC (2013). Adaptive immune responses during pregnancy. Am J Reprod Immunol.

[41] Polyzos NP, Polyzos IP, Zavos A, Valachis A, Mauri D, Papanikolaou EG (2010).

[42] Iheozor-Ejiofor Z, Middleton P, Esposito M, Glenny AM (2017). Treating periodontal disease for preventing adverse birth outcomes in pregnant women. Cochrane Database Syst Rev.

[43] Romero R, Miranda J, Kusanovic JP, Chaiworapongsa T, Chaemsaithong P, Martinez A (2015). Clinical chorioamnionitis at term I: microbiology of the amniotic cavity using cultivation and molecular techniques. J Perinat Med.

[44] Pierce BL, Ahsan H, Vanderweele TJ (2011). Power and instrument strength requirements for Mendelian randomization studies using multiple genetic variants. Int J Epidemiol.

