# Supplementary material for: Maternal periodontitis may cause lower birth weight in children: genetic evidence from a comprehensive Mendelian randomization study on periodontitis and pregnancy
Source: Clin Oral Investig. 2024 Mar 5;28(3):194. doi: 10.1007/s00784-024-05591-9 (PMC10914849; doi:10.1007/s00784-024-05591-9)
Supplement: Supplementary file 1 — Supplementary Material 1 [file 784_2024_5591_MOESM1_ESM.docx]

| SNP | Effect allele | Other allele  F-statistics for each selected SNP | Sample size | Beta | EAF | MAF | se | pval | R^2^ | F |
| --- | --- | --- | --- | --- | --- | --- | --- | --- | --- | --- |
| rs10268587 | G | A | 259234 | -0.29468 | 0.036863 | 0.036863 | 0.06212 | 2.10 × 10^-06^ | 0.006166 | 89.34752 |
| rs113059383 | G | A | 259234 | -0.83987 | 0.006079 | 0.006079 | 0.179505 | 2.88 × 10^-06^ | 0.008525 | 123.8173 |
| rs1148464 | T | C | 259234 | -0.14301 | 0.866449 | 0.133551 | 0.030727 | 3.25 × 10^-06^ | 0.004733 | 68.48781 |
| rs115120340 | T | C | 259234 | 0.184889 | 0.091773 | 0.091773 | 0.036091 | 3.01 × 10^-07^ | 0.005699 | 82.53381 |
| rs11605185 | C | T | 259234 | -0.23119 | 0.061205 | 0.061205 | 0.047486 | 1.12 × 10^-06^ | 0.006142 | 89.00023 |
| rs1241497 | A | G | 259234 | -0.12753 | 0.816954 | 0.183046 | 0.027534 | 3.63 × 10^-06^ | 0.004864 | 70.39222 |
| rs130985 | T | C | 259234 | -0.31308 | 0.029721 | 0.029721 | 0.068439 | 4.77 × 10^-06^ | 0.005653 | 81.8767 |
| rs139232605 | G | A | 259234 | 0.221301 | 0.061807 | 0.061807 | 0.041721 | 1.13 × 10^-07^ | 0.00568 | 82.25985 |
| rs141098993 | A | G | 259234 | 0.268631 | 0.031556 | 0.031556 | 0.056798 | 2.25 × 10^-06^ | 0.004411 | 63.79825 |
| rs146734691 | A | G | 259234 | 0.733419 | 0.002764 | 0.002764 | 0.160408 | 4.83 × 10^-06^ | 0.002966 | 42.83352 |
| rs2220232 | A | T | 259234 | 0.103497 | 0.454101 | 0.454101 | 0.02148 | 1.45 × 10^-06^ | 0.005311 | 76.88656 |
| rs2847728 | T | G | 259234 | -0.12594 | 0.210785 | 0.210785 | 0.027086 | 3.33 × 10^-06^ | 0.005277 | 76.39606 |
| rs35813112 | G | A | 259234 | 0.235956 | 0.042846 | 0.042846 | 0.049403 | 1.79 × 10^-06^ | 0.004567 | 66.06319 |
| rs55875437 | T | C | 259234 | 0.125636 | 0.203118 | 0.203118 | 0.02611 | 1.50 × 10^-06^ | 0.00511 | 73.96276 |
| rs56265851 | T | A | 259234 | 0.236029 | 0.044315 | 0.044315 | 0.049228 | 1.63 × 10^-06^ | 0.004719 | 68.27585 |
| rs6845106 | T | C | 259234 | -0.10165 | 0.59251 | 0.40749 | 0.02177 | 3.02 × 10^-06^ | 0.00499 | 72.21476 |
| rs7629105 | A | G | 259234 | -0.18919 | 0.933376 | 0.066624 | 0.040656 | 3.26 × 10^-06^ | 0.004452 | 64.39509 |
| rs9645299 | C | T | 259234 | 0.110372 | 0.713102 | 0.286898 | 0.024162 | 4.92 × 10^-06^ | 0.004985 | 72.14144 |

Statement: rs2220232, regarded as ambiguous and a palindrome by the harmonize function, is excluded from the following research.
