# Supplementary material for: Maternal periodontitis may cause lower birth weight in children: genetic evidence from a comprehensive Mendelian randomization study on periodontitis and pregnancy
Source: Clin Oral Investig. 2024 Mar 5;28(3):194. doi: 10.1007/s00784-024-05591-9 (PMC10914849; doi:10.1007/s00784-024-05591-9)
Supplement: Supplementary file 3 — Supplementary Material 3 [file 784_2024_5591_MOESM3_ESM.pdf]

Summarized plots of leave-one-out analysis

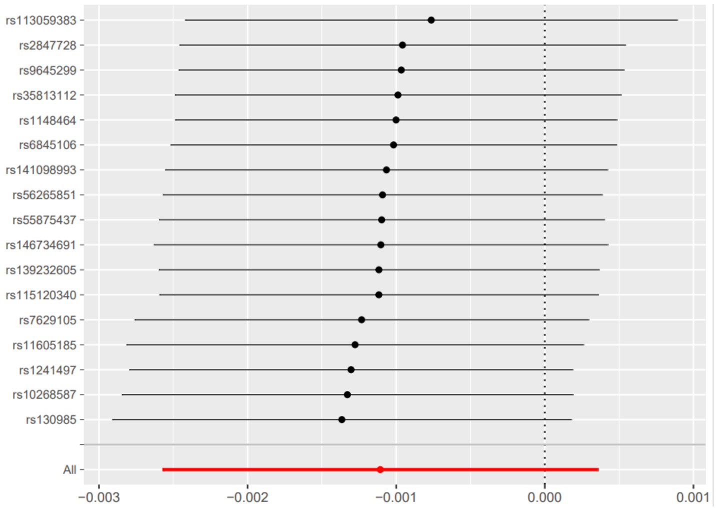

Fig 1. Excessive, frequent and irregular menstruation

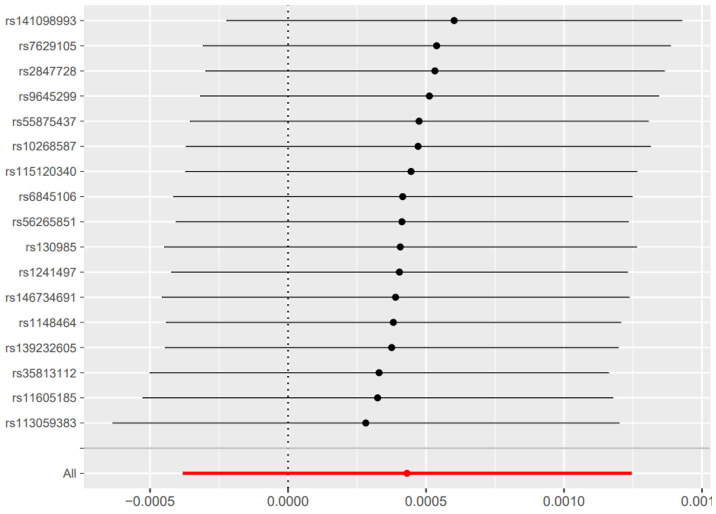

Fig 2. Abnormal uterine and vaginal bleeding

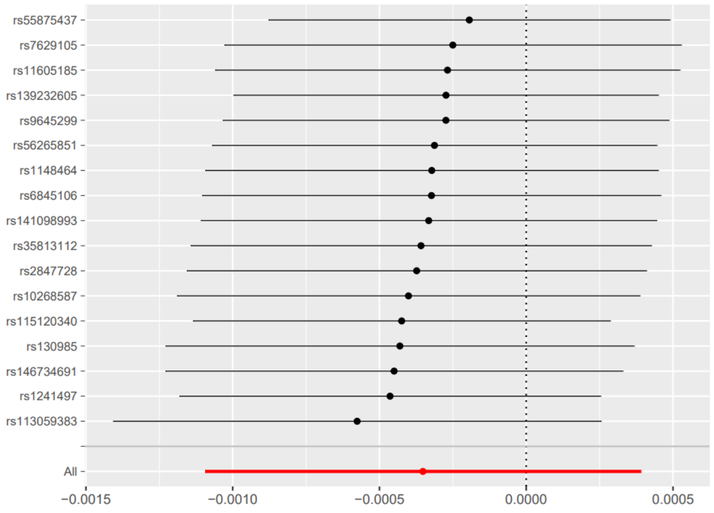

Fig 3. Endometriosis

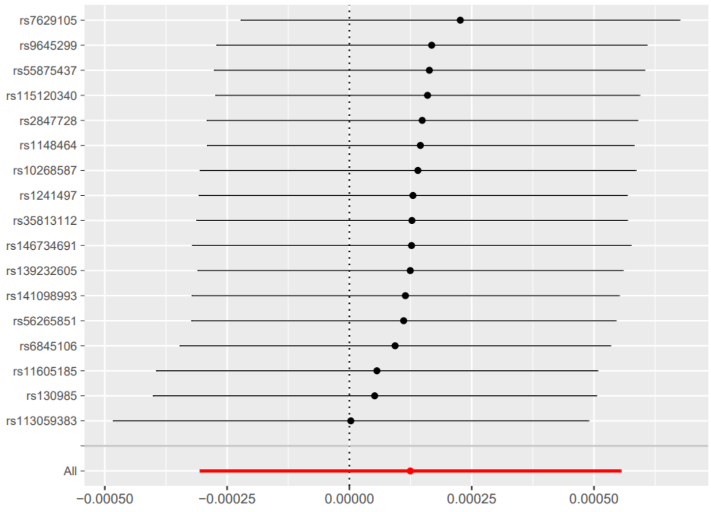

Fig 4. Female infertility

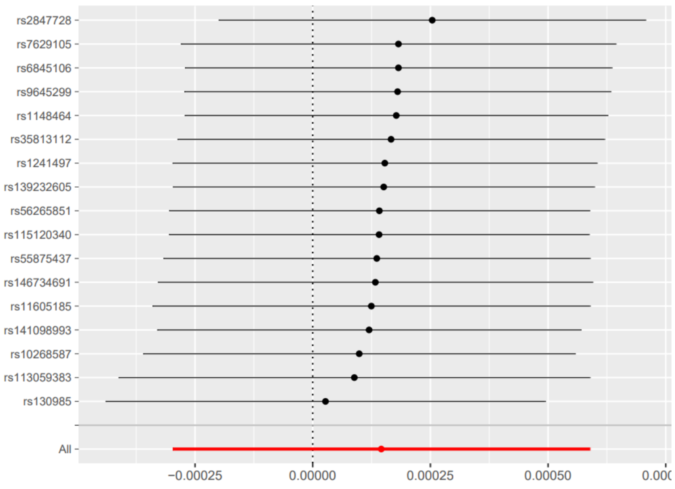

Fig 5. Hemorrhage in early pregnancy

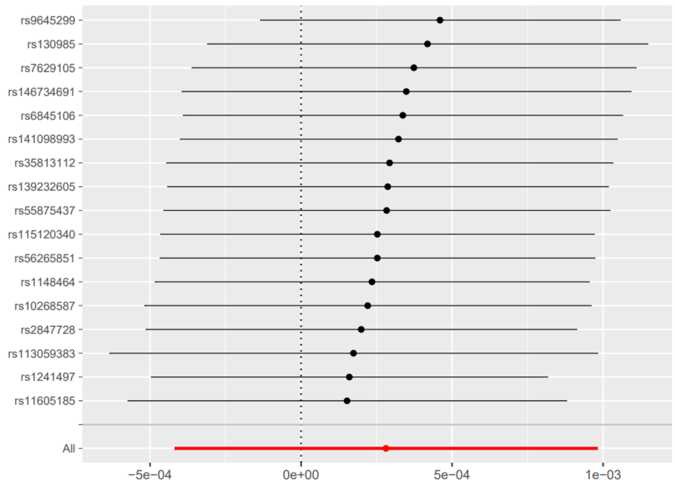

Fig 6. Other abnormal products of conception

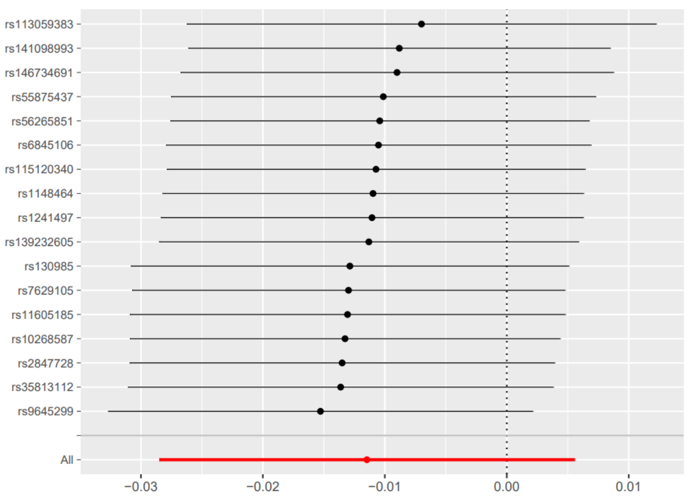

Fig 7. Number of spontaneous miscarriages

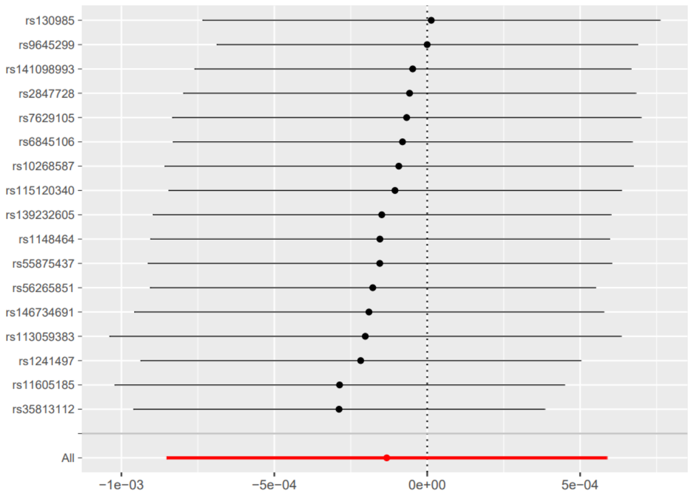

Fig 8. Single spontaneous delivery

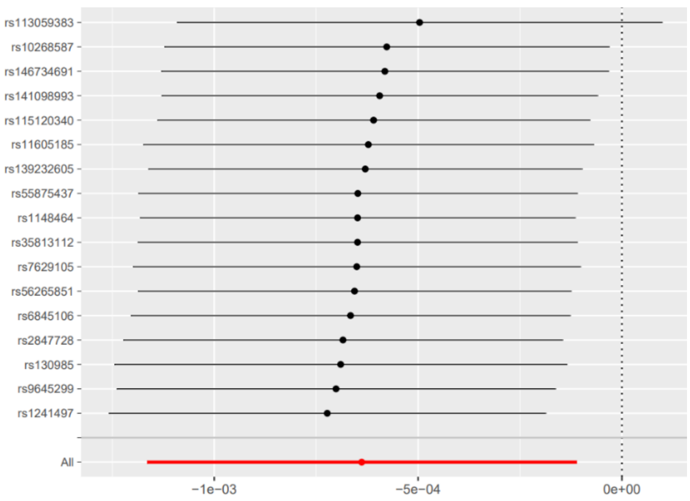

Fig 9. Long labour

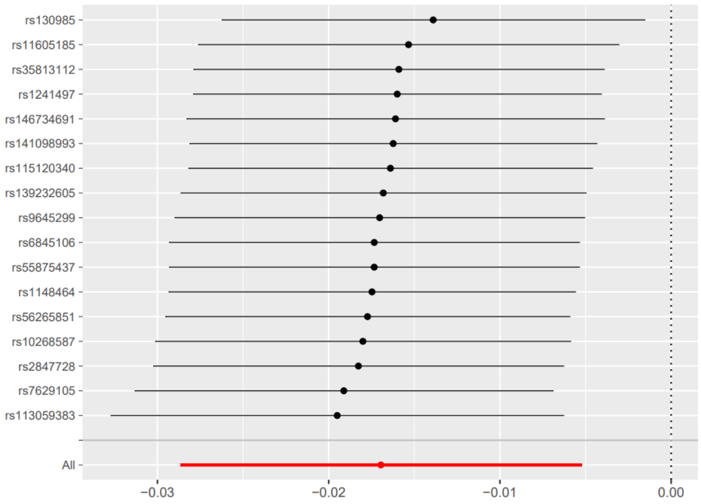

Fig 10. Birth weight of the first child
